# Metagenomic analysis of the lung microbiome in pulmonary tuberculosis - a pilot study

**DOI:** 10.1080/22221751.2020.1783188

**Published:** 2020-07-02

**Authors:** Yongfeng Hu, Min Cheng, Bo Liu, Jie Dong, Lilian Sun, Jian Yang, Fan Yang, Xinchun Chen, Qi Jin

**Affiliations:** a NHC Key Laboratory of Systems Biology of Pathogens, Institute of Pathogen Biology, CAMS&PUMC, Beijing, P. R. People’s Republic of China; b China Institute of Veterinary Drug Control, Beijing, People’s Republic of China; c Department of Pathogen Biology, Shenzhen University School of Medicine, Shenzhen, People’s Republic of China

**Keywords:** Lung microbiome, tuberculosis, metagenomic analysis, 16S rDNA, bronchoalveolar lavage

## Abstract

The lung microbiome plays an important role in the pathophysiological processes associated with pulmonary tuberculosis (PTB). However, only a few studies using 16S rDNA amplicon sequencing have been reported, and the interactions between *Mycobacterium tuberculosis* (MTB) and the lung microbiome remain poorly understood. Patients with respiratory symptoms and imaging abnormalities compatible with tuberculosis (TB) were enrolled. We analyzed the lung microbiome in bronchoalveolar lavage (BAL) samples from 30 MTB-positive (MTB+) subjects and 30 MTB negative (MTB-) subjects by shotgun metagenomic sequencing. Alpha diversity tended to be lower in the MTB+ group than in the MTB- group. There was a significant difference in *beta* diversity between the MTB+ and MTB- subjects. MTB+ lung samples were dominated by MTB, while MTB- samples were enriched with *Streptococcus*, *Prevotella*, *Nesseria*, *Selenomonas* and *Bifidobacterium*, which more closely resemble the microbial composition of a healthy lung*.* Network analysis suggested that MTB could greatly impact the microbial community structure. MTB+ and MTB- communities showed distinct functional signatures. Fungal communities were also found to be associated with the presence or absence of MTB. Furthermore, it was confirmed that 16S rDNA amplicon sequencing underrepresents *Mycobacterium*. This pilot study is the first to explore the interplay between MTB and the host microbiome by using metagenomic sequencing. MTB dominates the lung microbiome of MTB+ subjects, while MTB- subjects have a *Streptococcus*-enriched microbiome. The 16S approach underrepresents *Mycobacterium* and is not the best way to study the TB-associated microbiome.

## Introduction

Due to the development of shotgun and targeted metagenomic next generation sequencing, an increasing number of studies have revealed that the lung microbiome plays an important role in the pathophysiological processes associated with respiratory diseases [1]. The lungs are not sterile or free from bacteria; the dominant bacterial genera found in the lower airways are *Prevotella*, *Veillonella*, and *Streptococcus* during health [2]. Acute and chronic lung diseases can change the ecological determinants of the lung microbiome-immigration, elimination and regional growth conditions, resulting in markedly different microbial communities [3]. A better understanding of the nature and impact of the lung microbiome during health and disease may provide important information for diagnostic and/or therapeutic approaches.

Tuberculosis (TB), caused by the *Mycobacterium tuberculosis* (MTB) complex, is one of the top 10 causes of death and ranks as the leading cause from a single infectious agent worldwide with 10 million cases each year and 1.6 million deaths in 2017 [4]. MTB infection is initiated by inhalation of aerosol droplets carrying the bacilli. Alterations in the lung microbiome have been observed in many respiratory diseases, including TB, nontuberculous *Mycobacterium* (NTM) pneumonia, chronic obstructive pulmonary disease (COPD), asthma, and cystic fibrosis [1,5–9.] However, the lung microbiome in TB remains largely undefined. Very few studies by means of 16S rDNA sequencing have been reported to investigate the airway microbiome in TB patients with inconsistent and sometimes contradictory results [5]. It was also reported that *Mycobacterium* was frequently not identified using 16S rDNA sequencing even in samples with positive cultures for these organisms. In addition, 20% to 30% of patients with TB are diagnosed clinically due to lack of culture confirmation [10]. To date, the lung microbiome in MTB-positive (MTB+) and MTB-negative (MTB-) lung samples has not been analyzed. This pilot study is the first shotgun metagenomic analysis of the lung microbiome in bronchoalveolar lavage fluid (BALF) with or without MTB to investigate whether MTB is associated with a distinct lung microbiome.

## Materials and methods

### Study subjects and sampling

We enrolled subjects with respiratory symptoms and imaging abnormalities compatible with TB disease admitted to Shenzhen Third People’s Hospital in China. Patients underwent two sputum acid-fast bacillus smear examinations to diagnose PTB. Acid-fast bacillus smear-and culture-negative patients underwent bronchoscopy with bronchoalveolar lavage (BAL) for clinical diagnosis, with 1 mL set aside for microbiome analysis. MTB+ TB was defined as having a positive detection at least for *M. tuberculosis* on BALF by smear, culture, RT–PCR or the GeneXpert method. MTB- TB was regarded as having negative results on BALF by smear, culture and the GeneXpert, while clinical or radiographic improvement with anti-tuberculous treatment following the diagnostic guidelines recommendations [11]. Bronchoscopy was performed a median of 3 d after hospital admission (interquartile range, 1–4 d). All subjects had no immunosuppressive medications and negative for human immunodeficiency virus negative. Finally, residual aliquots (1 mL) of BALF were obtained from 60 subjects (30 MTB+ and 30 MTB- subjects) (Supplementary Table S1).

### Experimental design and DNA isolation

To obtain enough DNA for shotgun metagenomic sequencing, BALF from each patient was processed in pools of 5 (Supplementary Table S1). Each sample pool was centrifuged at 10,000×g for 30 min at 4°C. The pellets were resuspended in 500 µL of Hanks’ balanced salt solution, and DNA was isolated using a method combining homogenization and chemical lysis of cells with the QIAamp DNA Microbiome Kit (Qiagen). A pure culture of *Shigella flexneri* Sf301(equating to 10^5^ cells as input) was used as a negative control (subject to “blank” DNA extractions) and sequenced concurrently.

### Microbiome analysis

In brief, microbial DNA from 12 pools of samples (MTB+, 6; MTB-, 6) was processed without amplification, and barcoded libraries for multiplex high-throughput sequencing were constructed with the Nextera XT DNA Library Prep Kit following the manufacturer’s recommendations and sequenced on an Illumina HiSeq 2500 platform (Illumina, single-end, 125-bp read length). In parallel, the V3–V4 hypervariable regions of the bacterial 16S rDNA gene in the 12 pools were amplified using the multiplex barcoded primers 341F (CCTAYGGGRBGCASCAG) and 806R (GGACTACNNGGGTATCTAAT) and sequenced using an Ion S5 next-generation sequencing system.

### Sequence data processing

The raw metagenomic data were first filtered by base quality score and read length using Trimmomatic (v0.35; SLIDINGINDOW: 4:10 MINLEN: 70) [12]. Human sequences were discarded by Bowtie2 (v2.2.6-end-to-end, -sensitive) [13]. The remaining nonhuman reads were assembled and analyzed by SOAP *de novo* software (v2.04, -d 1, -M 3, -R, -u, -F, -K 55) [14]. Assembled and unassembled reads were directly mapped against the NCBI nt database (download date, May 20th, 2019) using BLASTN (v2.3.0, -task megablast, -evalue 1e-10, -max_target_seqs 10, -max_hsp 1, –qcov_hsp_perc 60) [15]. The results were then used as the input for MEGAN 6 (min score 100, top percent: 10), and the taxonomic assignment for each read was inferred using the lowest common ancestor (LCA) method [16]. Meanwhile, nonhuman non-rRNA reads were also mapped to the NCBI nr database using Diamond (v0.7.11, –sensitive –c 1) [17], with the thresholds used in MEGAN6 modified accordingly (min score: 40, max expected 0.001). The conversion file from Gi number to Kyoto Encyclopedia of Gene and Genomes (KEGG) was used to annotate the function of microbial reads [18]. In addition, the HMP Unified Metabolic Analysis Network (HUMAnN2) was used to determine the metabolic contributions within the samples. The HUMAnN2 pipeline involved mapping of the metagenomic reads against the KEGG database and MetaCyc pathway database [18–21]. As a pure culture was used as a negative control, any reads other than *S.flexneri* observed in subsequent DNA sequencing results were regarded as contamination and screened out of downstream analysis proportionally.

After Ion S5 sequencing, the individual sequence reads were filtered by PGM software to remove low quality and polyclonal sequences. Sequences matching the PGM 3′ adaptor were also automatically trimmed. All PGM quality-approved, trimmed and filtered data were exported as sff files. Singletons and low-abundance taxa (frequency < 10 considering all samples) were also discarded to reduce statistical noise, leaving the most abundant OTUs for downstream analyses (Supplementary methods). Metagenomic and 16S rDNA amplicon sequencing data are deposited at Sequence Read Archive under BioProject PRJNA580164.

### Statistical analysis

All statistical analyses were performed in the R environment and GraphPad Prism 7 (GraphPad Software, Inc., La Jolla, CA). Pearson’s chi-square test or Fisher’s exact test was used for categorical variables, and the Mann–Whitney U test or Kruskal–Wallis rank sum test was used for continuous variables that did not follow a normal distribution. Alpha diversity measures were compared using the Wilcoxon signed-rank test. An unsupervised ordination data-visualization technique (nonmetric multidimensional scaling, NMDS) was used to compare the overall structure (beta diversity). Permutational multivariate analysis of variance (PERMANOVA) with 1,000 permutations was performed, and the resulting *R*^2^ provided the proportion of variation explained. For comparison of the relative abundance of the most frequent phyla and genera between both groups, a Mann–Whitney U test was also performed using pairwise multiple comparison adjustments according to the Benjamini-Hochberg procedure. The correlation between two genera was considered statistically robust if the Spearman correlation coefficient (ρ) was >0.6 and the Benjamini-Hochberg adjusted *p*-value was <0.05. Statistical analyses were conducted by “psych” packages in R. The networks were visualized by Gephi (v0.9.2) (Supplementary methods).

## Results

### Overview

In total, the lung microbiome was examined in 12 BALF pools (5 BALF samples/each) from 60 active PTB subjects by shotgun metagenomic sequencing. After demultiplexing and stringent quality control filtering, a total of 584,381 reads were generated with an average of 48,698 ± 36,976 reads (mean ± SD) per pool, among which 92% could be assigned to a specific genus and 63% were assigned to a specific species or subspecies. Singletons and low-abundance taxa (frequency < 10 considering all samples) were discarded. Alpha and beta diversity analyses were performed using rarefied counts based on the lowest number of reads obtained across all samples (12,446 reads) (Supplementary Figure S1).

Overall, the dominant phyla were *Actinobacteria, Firmicutes, Proteobacteria, Bacteroidetes* and *Fusobacteria*, which were detected in all 12 samples and accounted for 99.6% of the total reads (Supplementary Figure S2). Of the 359 genera identified, *Mycobacterium, Streptococcus, Rothia, Actinomyces, Staphylococcus* and *Pseudomonas* were the most abundant; these genera accounted for 78.6% of the total reads. *Mycobacterium* was detected in all samples regardless of MTB+ or MTB– BALF samples but was dominant only in MTB+ samples.

### MTB+ or MTB- defines the lung community types

**Figure 1.**
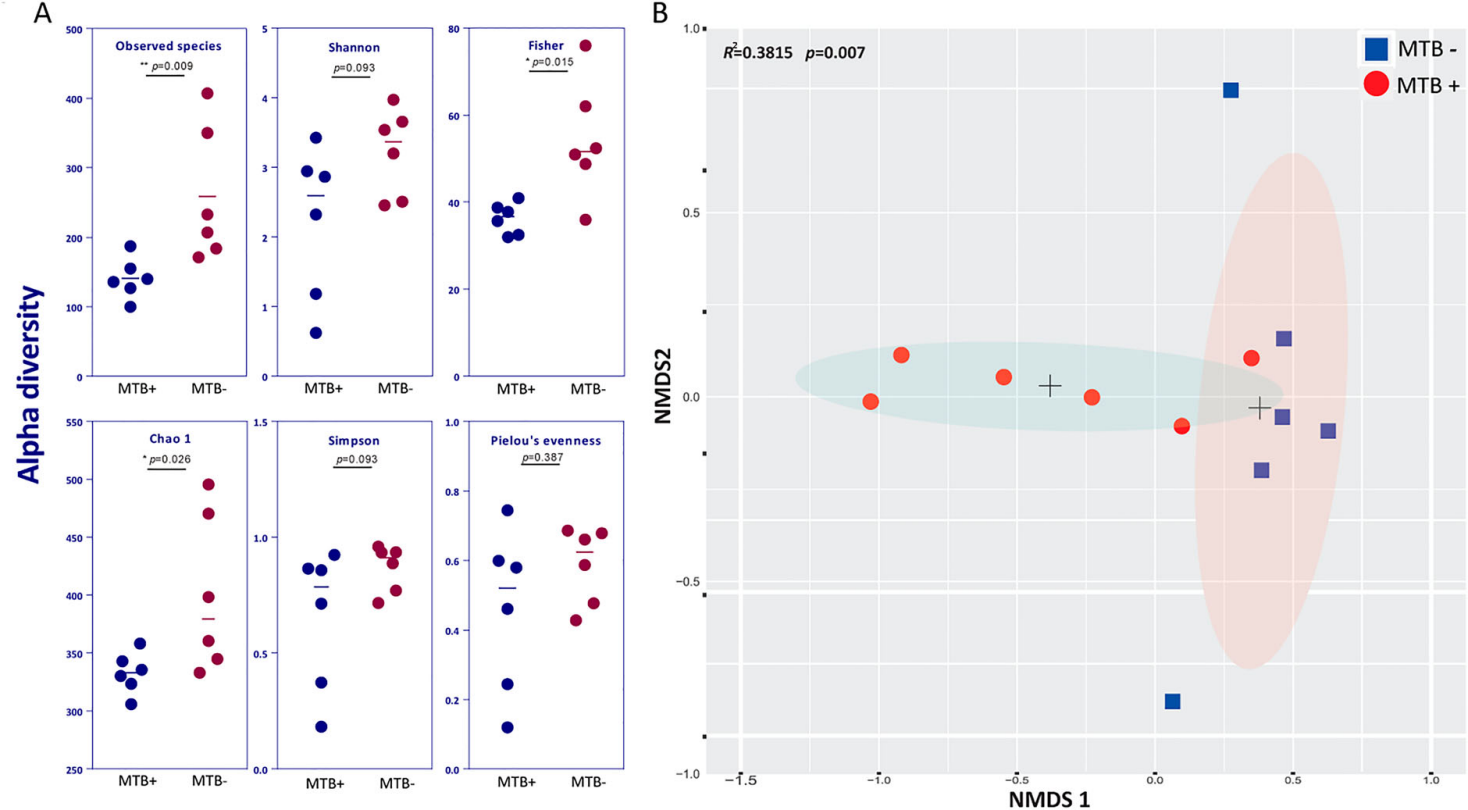
Alpha and beta diversity differences between MTB+ and MTB- patients. (A) Alpha diversity was calculated by the observed species, Shannon, Fisher, Simpson, Chao1 and Pielou's evenness indices. Significance was confirmed using the Mann-Whitney U test in GraphPad, Prism 7. (B) Beta diversity of lung bacterial communities in MTB+ and MTB- BALF specimens using unsupervised ordination (NMDS). Centroids are indicated by crosses. NMDS analysis demonstrated significant differences between the MTB+ and MTB- patients (PERMANOVA, *R*^2^ = 0.3815, *P* = 0.007).

Compared to MTB- patients, MTB+ patients showed a significantly reduced alpha diversity (microbial diversity within a sample), as calculated by the observed species, Fisher and Chao1 indices (Figure 1A, *P* < 0.05). The distinct separation of the two centroids in NMDS analysis demonstrated a significant difference in average community composition between MTB+ and MTB- patients (Figure 1B, Adonis, Bray–Curtis dissimilarity, *R*^2^ = 0.3815, *P* = 0.007). Removal of the dominant genus (*Mycobacterium*) reads and reapplication of NMDS to the remaining data yielded similar results, indicating that *Mycobacterium* is not the sole defining feature of the airway microbiome (Supplementary Figure S3).

**Figure 2.**
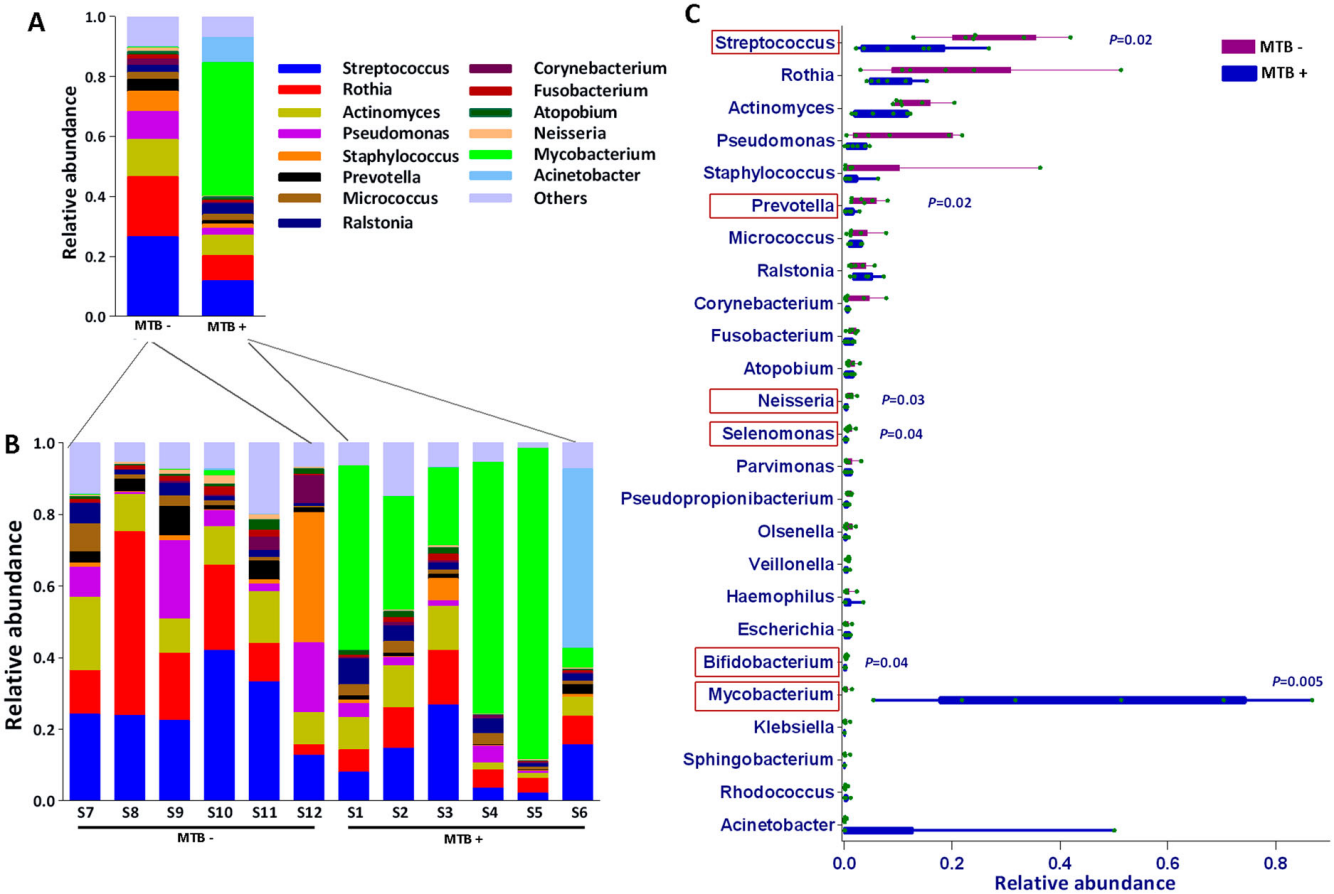
Average relative taxon abundance comparisons between the MTB+ and MTB- groups at the genus level. *P*-values were determined using the Mann-Whitney U test, and the Benjamini-Hochberg procedure (false discovery rate correction method) was applied to obtain adjusted *p* values for multiple comparisons between groups. Boxes indicate 5th to 95th percentiles, with median relative abundances marked as lines and whiskers indicating the range (minimum/maximum) multiplied by the interquartile range (5th to 95th percentiles) from the boxes. Bacterial taxa are ranked by average relative abundances of the of overall lung microbiome in MTB- patients.

The relative abundances of the taxa at different taxonomic levels were further analyzed. Phylum-level analysis showed that the relative abundance of *Actinobacteria* in MTB+ patients was obviously (but not significantly, *P* = 0.06, Wilcoxon test) higher than that in MTB- patients. This result was accompanied by a lower relative abundance of *Firmicutes* (*P* = 0.012, Wilcoxon test) and *Bacteroidetes* (*P* = 0.030, Wilcoxon test) (Supplementary Figure S2). At the genus level, *Mycobacterium* was significantly enriched in MTB+ subjects (*P* = 0.005, Wilcoxon test; abundance, 5.4–86.8%, average 44.6% *vs* 0.02–1.4%, average 0.3%), while *Streptococcus, Prevotella, Neisseria, Selenomonas* and *Bifidobacterium* were significantly enriched in MTB- subjects, which more closely resembled the microbiome composition of the healthy lung, as reviewed by a previous study [2,3] (Figure 2A-C). Further taxonomic breakdown (species level) revealed that 77.9% of *Mycobacterium* sequences corresponded to MTB in MTB+ subjects, which dominated the lung microbiome. *Pseudomonas aeruginosa* and *Streptococcus salivarius* were enriched in MTB- samples (Supplementary Figure S4).

### Network analysis reveals potential microbial interactions

**Figure 3.**
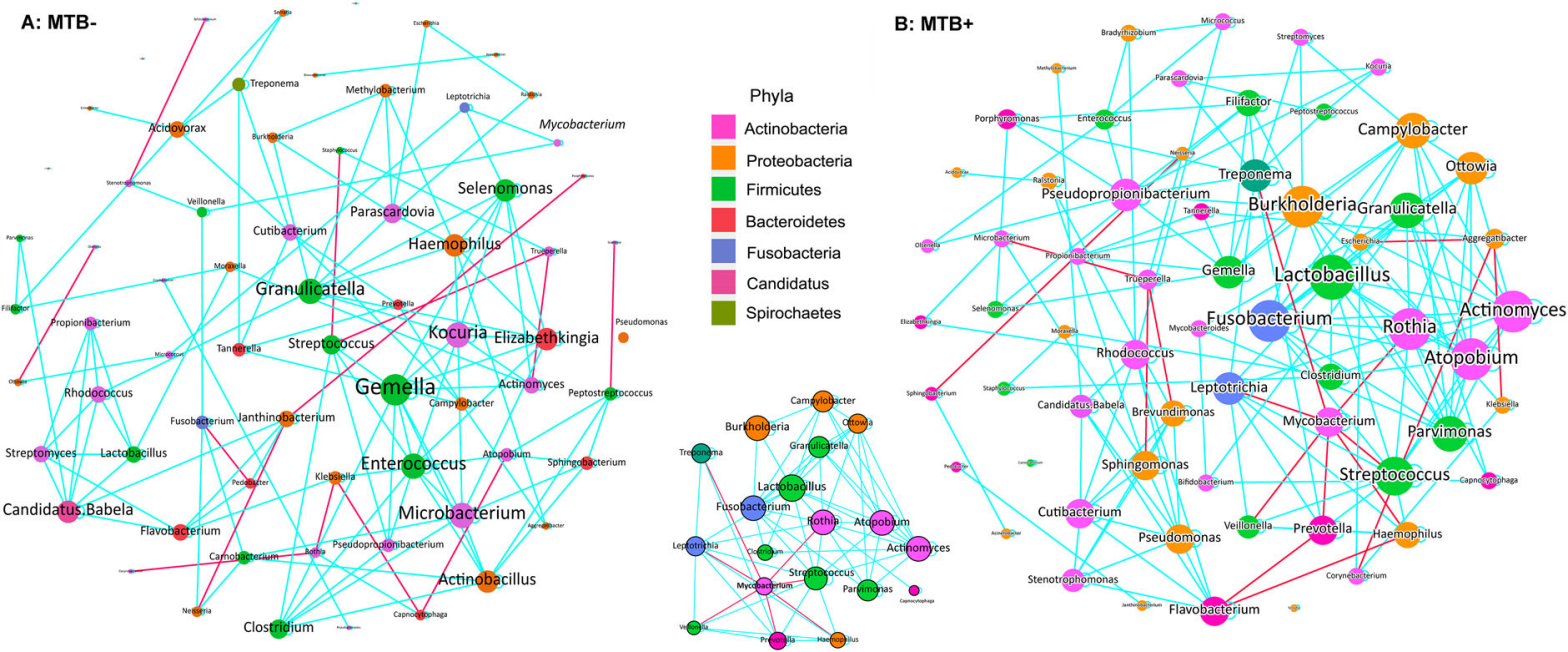
Co-occurring network of microbial communities in lung samples from MTB+ and MTB- patients based on correlation analysis. The connections in the network represent a strong (*ρ* > 0.6) and significant (*P* < 0.05) correlations. The nodes are colored by phylum. The size of each node is proportional to the number of connections. The thickness of each edge is proportional to the ρ. Light blue lines represent positive correlations, and purple lines represent negative correlations.

The specific interactions of MTB and the lung microbiome during infection are entirely unexplored. We compared the co-occurrence of taxa between the MTB+ and MTB- groups to determine how specific microbes potentially interact with each other in the community and how their interactions are disrupted by MTB infection. A co-occurring network containing strong (*ρ* >  0.6) and significant (FDR-adjusted *P* < 0.05) correlations between 68 bacterial genera was constructed. Overall, the total number of microbial interactions, as indicated by the number of edges between the nodes, showed no difference between MTB+ and MTB- groups (MTB+: *n* = 153, MTB-: *n* = 123, chi-square, *P* = 0.41). *Mycobacterium*, which had only 3 connections (all positive) in MTB- samples, established 7 negative connections with other members of the microbiome in MTB+ samples (Figure 3A and B). This finding implied that MTB may compete with specific taxa and affect the overall network. Examination of the microbial network revealed that it was predominated by a few “hub” genera that were highly connected with multiple other nodes, and the MTB may change the hub from one to another. For example, *Rothia* had only 3 connections (2 negative, 1 positive) in the MTB- group, while the genus had a high degree of connectivity in the MTB+ group network, with a co-exclusive relationship with MTB and a co- existence relationship with 11 other genera. We also observed that *Pseudomonas* established 8 new taxonomic interactions and became a central node in MTB+ subjects, while it was independent of other taxa in MTB- subjects (Figure 3A and B).

### MTB+ and MTB- communities show distinct functional signatures

**Figure 4.**
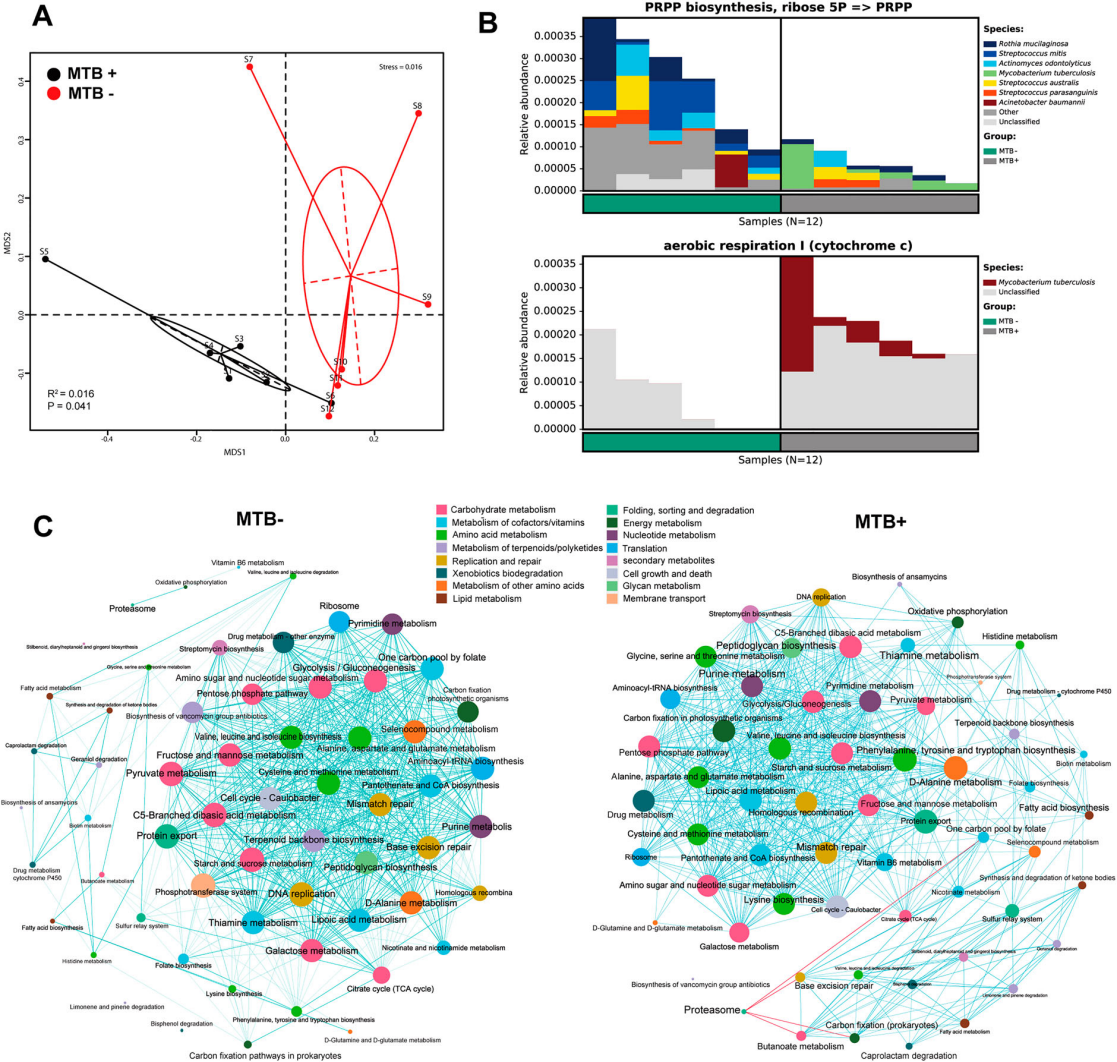
MTB+ and MTB- lung community are functionally distinct. A: a NMDS plot of Bray–Curtis resemblance generated from the square root-transformed KEGG pathway (level 3) relative abundances (generated using HUMAnN2); B: Functional differences between the MTB+ and MTB- patients based on selected metabolic pathways and bacteria associated with such functions through read-mapping; C: network analysis of microbial metabolic profiles in lung samples from MTB+ and MTB- patients based on correlation analysis. The connections in the network represent a strong (*ρ* > 0.6) and significant (*P* < 0.05) correlations. The nodes are coloured by KEGG pathway (level 2). The size of each node is proportional to the number of connections. Light blue lines represent positive correlations, and red lines represent negative correlations.

To explore differences in the metabolic potential of the lung microbiome between MTB+ and MTB- patients, we further estimated the abundance of metabolic pathways using our metagenomic reads mapped to functional orthologs from the KEGG and MetaCyc databases with the HUMANn2 pipeline (Supplementary Table S2). The taxonomic community types were functionally different across KEGG and MetaCyc (Figure 4A, PERMANOVA *R*^2^ = 0.016, *P* = 0.041). We identified a significantly decreased gene abundance for PRPP biosynthesis in MTB+ patients compared to that in MTB- patients (system: carbohydrate metabolism; central carbohydrate metabolism; PRPP biosynthesis, ribose 5P ≥ PRPP, KEGG module number M00005, Wilcoxon t-test, *P* = 0.004, Figure 4B). This result was corroborated by a decreased abundance of genes in the pentose phosphate and purine metabolism pathways (KEGG pathway ko00030/ko00230, Wilcoxon test, *P* = 0.004/0.0087, respectively) (Supplementary Table S2). We also found one pathway for aerobic respiration I (cytochrome C, MetaCyc pathway number PWY-3781, Wilcoxon test, *P* = 0.004, Figure 4B), which appeared to be more active in the microbiome of MTB+ patients than in the microbiome of MTB- patients. To determine which bacteria are involved in these pathways, we traced the contributing genes and determined their likely taxonomic origin (Figure 4B). Although several species contribute to these pathways, we only found evidence that MTB contributed significantly more reads in MTB+ patients than MTB- patients in the aerobic respiratory I pathway, while in the PRPP biosynthesis module, Streptococcus contributed significantly more reads in MTB- patients than MTB+ patients (Figure 4B).

Furthermore, a network analysis of the functional profiles among the top 60 abundant KEGG pathways was performed to determine the metabolic co-occurrence patterns in both groups. Overall, the total number of microbial interactions showed no difference between MTB+ and MTB- samples (MTB-: *n* = 668, all positive; MTB+: 619, positive 615, negative 4; chi-square, *P* = 0.37). Interestingly, the *Proteasome* pathway, which had only 1 positive connection in MTB- communities, established 4 negative connections with other pathways, such as butanoate metabolism and carbon fixation, in MTB+ samples. Likewise, lysine biosynthesis, fatty acid biosynthesis, vitamin B6 and drug metabolism showed more co-occurrence with other pathways in MTB+ than in MTB-. Otherwise, one carbon pool by folate, thiamine and pyruvate metabolism were less connected with other pathways in MTB+ than in MTB- samples (Figure 4C).

### 16S rDNA amplicon sequencing underrepresents Mycobacteria

**Figure 5.**
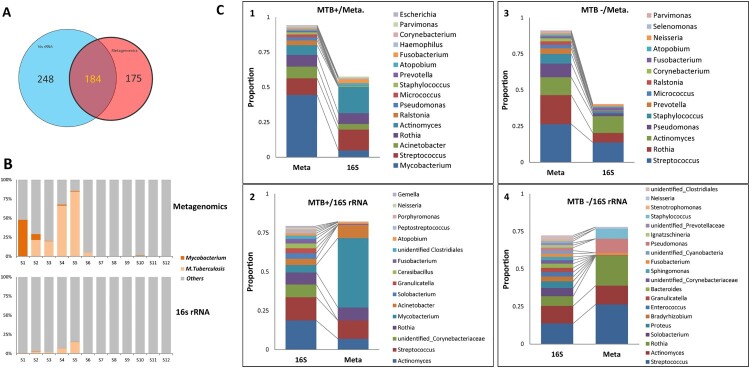
Comparison of microbial composition between the metagenomic and 16S rDNA data. A: Overlap of identified genera between two data sets. B: Comparison of the read abundance of *Mycobacteria* and *M. tuberculosis* in lung microbiome *from* MTB+ and MTB- patients revealed by the metagenomic and 16S rDNA gene amplicon sequencing. C: (1) the top 10 most abundant genera in the metagenomic data in MTB+ data sets; (2) the top 10 most abundant genera in the 16S rDNA data in MTB+ data sets; (3) the top 10 most abundant genera in the metagenomic data in MTB- data sets; (4) the top 10 most abundant genera in the 16S rDNA data in MTB- data sets.

16S rDNA sequencing data were successfully obtained for the 12 pools (V3–V4 region, with 28,320 reads each); 432 genera were identiﬁed, 184 (43%) of which were also identified in the metagenomic data (184/359, 51%), (Figure 5A). The 184 genera accounted for 76% of the 16S rDNA reads and 97% of the metagenomic reads, suggesting that the high-abundance genera could be identiﬁed by both methods. However, the relative abundances determined were not always comparable between the two methods. The metagenomic method detected more *Mycobacterium*, consisting mainly of *M. tuberculosis,* while 16S rDNA data obviously underrepresented the genus (Figure 5B and C). In the MTB+ group, *Mycobacterium* had the highest abundance in the metagenomic data, while *Actinomyces* and *Streptococcus* were more highly enriched in the 16S rDNA data (Figure 5C-1 and C-2); for the MTB- subjects, *Streptococcus*, *Actinomyces* and *Rothia* were the three most abundant genera in both the metagenomic and 16S rDNA data (Figure 5C-3 and C-4).

Furthermore, we compared microbial alpha and beta diversity in the 16S amplicon and metagenomic analysis. By measuring alpha diversity using the observed genus, Shannon and Simpson indices, we found that MTB- patients also had higher alpha diversity than MTB+ patients in 16S amplicon analysis, which is in line with the metagenomic analysis (Supplementary Figure S5A). Likewise, NMDS analysis showed that beta diversity was different between MTB- patients and MTB+ patients in 16S amplicon analysis (Supplementary Figure S5B). Our analysis showed that all of the alpha diversity indices in the 16S amplicon analysis were higher than those in the metagenomic analysis. Lung microbiome revealed by 16S rDNA sequencing tends to be more diverse, including more unidentified genera. This phenomenon is likely because the information provided by this method is limited due to its narrow detection spectrum (bacteria) and low resolution (genus). Our study demonstrated that the 16S rDNA sequencing approach significantly underrepresented *Mycobacteria*, which is in line with a previous study [6].

Bacterial metagenome content was also predicted from 16S rRNA gene-based microbial compositions, and functional inferences were made from the KEGG catalog using the PICRUSt algorithm. A total of 225,866,890 inferred genes were categorized into 328 KEGG functional pathways; pathways present in <10% of participants were removed, leaving 301 KEGG pathways (level 3) for analysis (Supplementary Table S3). The 25 most abundant functional pathways were related mainly to membrane transport, genetic information processing and metabolism, including pathways related to the ribosome, aminoacyl tRNA biosynthesis, glycolysis/gluconeogenesis, aspartate and glutamate metabolism and pyruvate metabolism, which were also among the top 25 KEGG pathways from the metagenomic analysis (Supplementary Table S3). Of the 301 KEGG pathways tested, 18 pathways (level 3) differed in abundance between MTB+ and MTB- patients (*P* < 0.05); these included pathways associated with Purine metabolism, Ribosome, Bacterial chemotaxis, ABC transporters, Pyrimidine metabolism, Aminoacyl-tRNA biosynthesis and Bacterial secretion systems, all of which were also significantly different between MTB+ and MTB- patients in metagenomic analysis (*P* < 0.05) (Supplementary Table S3).

Furthermore, we performed a comparison in network analysis between the 16S amplicon and metagenomic analysis. Overall, the total number of microbial interactions, as indicated by the number of edges between the nodes, showed no difference between MTB+ and MTB- samples (MTB-: *n* = 90, positive 61, negative 39; MTB+: *n* = 98, positive 52, negative 46). *Mycobacterium*, which had only 2 positive connections in MTB- samples, had 5 connections with other members of the microbiome in MTB+ samples, including a negative connection with *Solobacterium* (Supplementary Figure S5C). Additionally, several “hub” genera shifted as the MTB- communities shifted to MTB+ communities. For example, *Streptococcus* with a high degree of connectivity in the MTB- group had only 5 connections in the MTB+ group. Moreover, *Ignatzschineria* and *Bradyrhizobium* became the new “hub” genera in the MTB+ group (Supplementary Figure S5C).

### Fungal diversity

**Figure 6.**
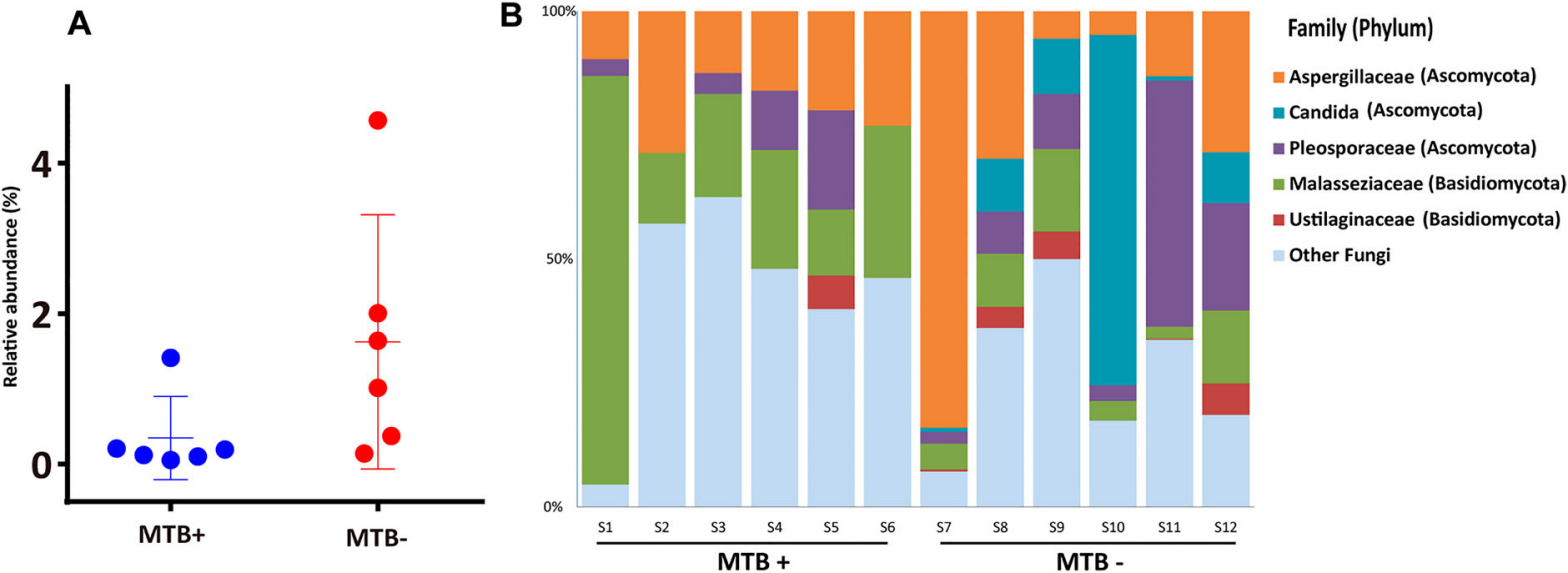
Phylum-level analysis of fungal sequences. A: Comparison of the relative abundance of fungal communities in lung microbiome from the MTB+ and MTB- patients; B: the relative abundance of fungal families in bronchoalveolar lavage fluid from each MTB+ and MTB- patient.

The reads assigned to fungi through BLASTN against the NCBI nt database (download date, May 20th, 2019) in each sample were analyzed further. Although with low relative abundance (range: 0.1–4.6%; median: 0.3%), the fungi in the lungs of MTB+ subjects were significantly less abundant than those in the lungs of MTB- subjects (Figure 6A, Wilcoxon test, *P* = 0.04; median: 0.2% *vs* 1.3%). Overall, the majority of the sequences analyzed were classified as belonging to the phylum *Ascomycota*, followed by *Basidiomycota* (Figure 6B). This result is consistent with the findings by means of ITS analysis by Botero and his colleagues, who demonstrated that *Aspergillus* and *Candida* were the most frequent genera in both sputum and oropharyngeal samples from TB patients [22]. In this study, *Aspergillaceae* and *Malasseziaceae* were detected in all samples, while *Candida* was found only in MTB- subjects*.* As we know, the majority of cases infected by fungi of the genus *Aspergillus* occurred in people with underlying illnesses such as TB or COPD, but with otherwise healthy immune systems. Previous work on skin microbial communities indicated that diversity was dependent on body site, while in this study, the fungal communities were found to be associated with the presence or absence of MTB [23].

## Discussion

This pilot study is the first to explore the lung microbial communities using shotgun metagenomic sequencing techniques in a prospective cohort of patients suspected of having PTB. As we know, only a few studies using 16S rDNA amplicon sequencing have investigated the lung microbial changes associated with TB. However, small sample sizes, limited sequencing depth, and sputum specimens pose significant limitations in the interpretation of the results. Thus, standardization of collection and sequencing methods, accounting for contamination, and consistent presentation of data and analysis are urgently needed. Our study, in line with a previous study [6], demonstrates that 16S rDNA sequencing significantly underrepresents the *Mycobacterium* genus. This phenomenon may occur for the following reasons: (1) more powerful methods may be required for thorough lysis of the mycobacterial cell wall to isolate genomic DNA due to the large amount of fatty acids and waxes in the cells [24]; and (2) because MTB tends to have only one or two copies per genome, 16S rDNA amplicon sequencing tends to underrepresent this genus. Thus, we suggest that an integrated analysis of the 16S rRNA gene and metagenomic sequencing may be needed to greatly advance our understanding of the lung microbiome associated with TB. Shotgun metagenomic sequencing has enabled more in-depth characterization and insights into the function of lung microbiomes than 16S rDNA amplicon sequenc­ing. However, obtaining enough DNA from lung samples for sequencing is challenging because the bacterial burden in the lung is approximately one million-fold lower than that in the gut and one hundred-fold lower than that in the upper airway [3]. In this study, BALF samples from each patient were processed in pools of 5 to obtain enough DNA for shotgun metagenomic sequencing. The results showed that the metagenomic method accurately revealed microbial diversity in both MTB+ and MTB- patients.

Culture-negative PTB is likely an early disease state on the continuum between MTB infection and disease, which, if left untreated, can advance to culture-positive disease [10]. It was reported that classic TB symptoms are not always associated with culture-negative disease, which had less hemoptysis, sputum production, weight loss, and cavitary lesions on chest computed tomography (CT) [10]. Our study showed that there was also a significantly different microbial community between MTB+ and MTB- subjects. The lung microbiome dominated by *Streptococcus* and *Prevotella* in MTB- subjects more closely resembled the healthy lung microbiome revealed by a previous study [2]. In this study, the majority of fungal sequences were classified as belonging to the phylum *Ascomycota*, followed by *Basidiomycota*. This result is consistent with the findings by means of ITS analysis by Botero [22].

In conclusion, this pilot study is the first to explore the lung microbial communities and their network and functional signatures associated with TB by metagenomic sequencing. MTB+ and MTB- lung microbial communities showed microbial diversity, distinct functional signatures and overall networks. The 16S rDNA sequencing underrepresents *Mycobacterium*, which is consistent with the observation in NTM cases.
